# A Novel Titanium End-Disc-Assisted Allograft Preparation System for Anterior Cervical Corpectomy and Fusion: Development and Preliminary Biomechanical Evaluation

**DOI:** 10.3390/bioengineering13080893

**Published:** 2026-08-03

**Authors:** Chih-Chang Chang, Shao-Fu Huang, Pin-Cang Huang, Chun-Li Lin

**Affiliations:** 1 Department of Biomedical Engineering, National Yang Ming Chiao Tung University, Taipei 112201, Taiwan; ccchang74@gmail.com (C.-C.C.);; 2 Department of Neurosurgery, Neurological Institute, Taipei Veterans General Hospital, Taipei 112201, Taiwan; 3 School of Medicine, National Yang Ming Chiao Tung University, Taipei 112304, Taiwan; 4 Medical Device Innovation & Translation Center, National Yang Ming Chiao Tung University, Taipei 112304, Taiwan

**Keywords:** anterior cervical corpectomy and fusion, allograft, titanium endplate, spinal implant, biomechanical testing, graft preparation, cervical reconstruction

## Abstract

**Background:** Allograft bone remains an attractive option for anterior cervical corpectomy and fusion (ACCF), but its use can be limited by variable graft geometry, insufficient endplate contact, and operator-dependent trimming. This study aimed to develop a titanium end-disc-assisted allograft construct with a standardized cutting and assembly system and to preliminarily evaluate its static mechanical integrity under axial compression, compression-shear, and torsional loading. **Methods:** The construct consisted of a controlled-length bone graft, two Ti6Al4V end-discs, and four countersunk fixation screws, forming a graft–disc construct with a maximum total height not exceeding 40 mm. The end-discs incorporated angled screw holes, bone-graft filling spaces, and enlarged contact surfaces. A modular preparation system was designed for graft clamping, length definition, guided cutting, alignment, pre-drilling, and screw fixation. Porcine rib specimens were used as an allograft substitute. Native bone and assembled constructs were tested under static compression, compression-shear, and torsion. **Results:** The system produced standardized constructs. Except for axial compressive stiffness, the assembled constructs showed numerically higher compressive yield force, shear stiffness, shear yield force, torsional stiffness, and yield torque than native bone; however, these findings were interpreted descriptively because of the limited sample size. Failure occurred mainly within the bone, with no obvious failure at the bone–titanium interface, titanium end-discs, or fixation screws. **Conclusions:** The proposed system enabled standardized graft preparation and construct assembly. Further fatigue, subsidence, and clinically relevant cervical spine model testing are required.

## 1. Introduction

Anterior cervical corpectomy and fusion (ACCF) is commonly performed to treat spinal cord or nerve root compression caused by multilevel cervical pathology, ossification of the posterior longitudinal ligament, severe intervertebral stenosis, and cervical deformity [1,2,3,4,5]. After removal of the diseased vertebral body and adjacent intervertebral disc tissues, reconstruction of the anterior column is required to restore cervical height, maintain sagittal alignment, provide immediate structural support, and facilitate subsequent bone fusion [6,7]. Therefore, establishing a reconstructive unit that provides adequate mechanical stability, stable endplate contact, and a favorable environment for bone fusion is critical to the success of ACCF.

Several options are currently available for anterior column reconstruction in ACCF, including autologous bone grafts, allografts, titanium mesh cages, and expandable cages; however, each strategy has inherent limitations [8,9,10,11,12,13]. Autologous bone grafts provide favorable osteogenic and fusion potential but require additional bone harvesting, which may prolong operative time and increase donor-site morbidity, including pain, infection, and limited graft availability [10,14]. Titanium mesh cages provide good structural strength and can be packed with bone graft or bone substitute materials. However, they usually require manual trimming to match the defect height. The trimmed edges may become sharp or uneven, resulting in insufficient endplate contact area and local stress concentration, thereby increasing the risk of subsidence [11,12,15,16,17]. Expandable cages allow intraoperative height adjustment and restoration of vertebral support, but their structures are more complex and costlier. In addition, inadequate fusion space or insufficient fixation stability may still lead to poor fusion, subsidence, or implant displacement [9,13]. Therefore, although existing metallic reconstruction devices can provide immediate mechanical support, they may not simultaneously address cost, fusion environment, end-face contact, and procedural simplicity.

In contrast, allografts remain an attractive option for ACCF reconstruction because their structural characteristics are closer to those of native bone tissue, they provide osteoconductive potential, and they avoid the additional surgical wounds and donor-site complications associated with autologous bone harvesting [10,14]. In addition, allografts are typically available from hospital or tissue donation bone banks and can be obtained in various sizes [14]. Compared with high-cost metallic cages or complex expandable devices, allografts may reduce implant-related costs in selected clinical settings, which is particularly relevant for patients with limited medical resources or substantial financial burden. Therefore, if the preparation, cutting, and fixation of allografts can be further improved, they may represent a biologically favorable and cost-effective option for ACCF reconstruction.

**Figure 1:**
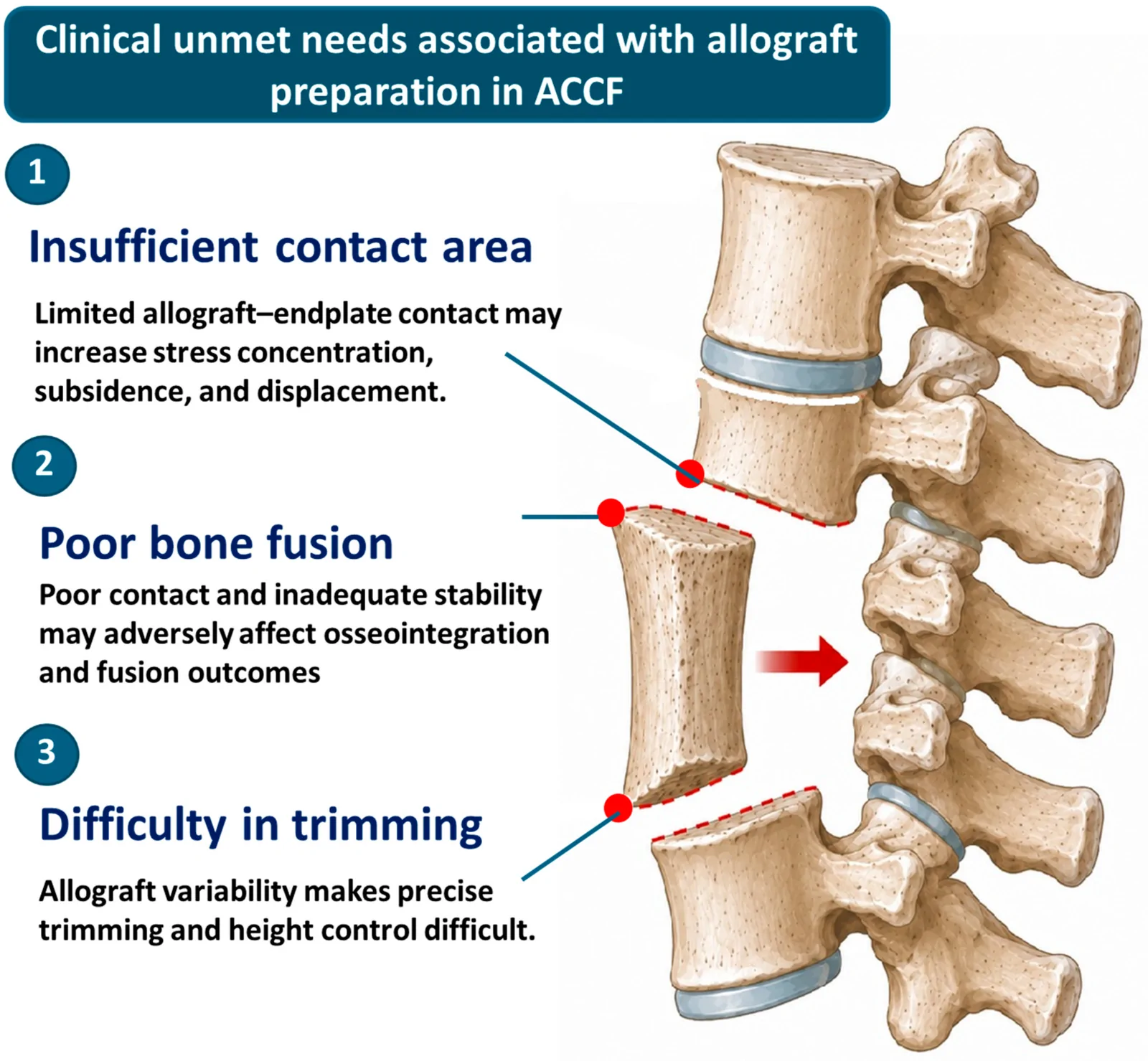
Clinical unmet needs associated with allograft preparation in ACCF. Conventional allograft preparation may result in insufficient graft–endplate contact area, limited fusion potential, and difficulty in achieving accurate trimming with flat cut surfaces. These operator-dependent factors may increase the risk of local stress concentration, graft instability, subsidence, displacement, or poor fusion.

However, allografts vary in source, shape, and size, and manual trimming is usually required before or during surgery to match the height of the vertebral defect. This process is highly dependent on the surgeon’s experience. Inaccurate trimming height, an uneven cut surface, or insufficient contact area between the graft and the adjacent vertebral endplates may lead to local stress concentration and contact instability, thereby increasing the risk of postoperative subsidence, displacement, or poor bone fusion [12,15,16,17,18] (Figure 1). Therefore, the use of allografts in ACCF still involves three unresolved clinical needs. First, the contact area between the allograft end surface and the vertebral endplate may be insufficient, causing the load to be concentrated in localized regions. Second, although allografts possess osteoconductive potential, the lack of stable fixation and adequate space for bone graft filling may still compromise fusion performance. Third, the graft height and cut-surface geometry must be adjusted by trimming; however, standardized tools for graft clamping, length (high) definition, and cutting guidance are currently lacking, making the preparation outcome susceptible to operator-dependent variability [14,18].

To address these issues, this study proposed a modular titanium end-disc fixation and cutting-guidance system for allograft preparation in ACCF. The system consisted of proximal and distal titanium end-discs, countersunk fixation screws, bone-graft filling spaces, and auxiliary instruments designed to control allograft length and improve cut-surface flatness. The design aimed to standardize the allograft preparation process, increase the contact area between the allograft and vertebral endplates, preserve potential pathways for bone-graft contact and fusion, and improve the initial structural stability of the assembled reconstruction unit. Preliminary static biomechanical tests were conducted using independent porcine rib specimens to evaluate the feasibility of the design as an auxiliary system for allograft-based ACCF reconstruction.

## 2. Materials and Methods

### 2.1. Design of the Titanium Graft–Disc Construct

**Figure 2:**
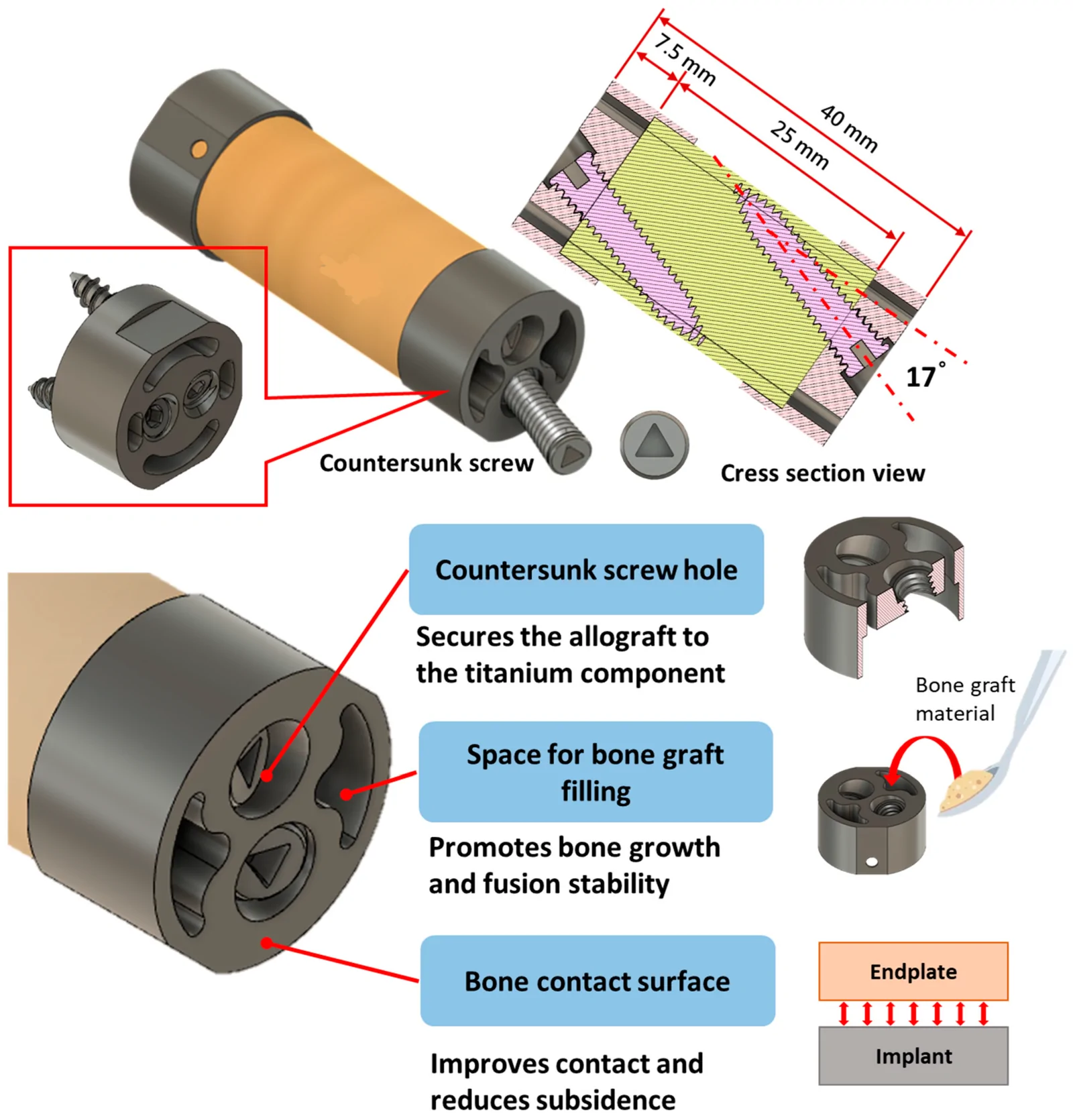
Design concept of the titanium end-disc-assisted allograft construct. The construct consists of an allograft segment, proximal and distal titanium end-discs, and fixation screws. The titanium end-disc incorporates countersunk screw holes, spaces for bone graft filling, and an enlarged bone-contact surface to improve construct fixation, preserve fusion space, and increase graft–endplate contact.

This study proposed a titanium end-disc-assisted allograft fixation construct for ACCF. The design concept involved securing titanium end-discs to both the proximal and distal ends of the allograft to increase the contact area between the graft and the superior and inferior vertebral endplates. The end-discs also provided open internal spaces for bone graft or bone filler, thereby preserving potential fusion pathways. The complete construct consisted of one allograft segment, two titanium end-discs, and four fixation screws. Only the titanium end-discs and fixation screws were intended to remain in the patient as implantable components; the clamping, cutting, alignment, and drilling modules were designed as extracorporeal preparation instruments. Based on the requirements for anterior column reconstruction after a two-vertebral-body corpectomy defect, the test specification was defined as a maximum total construct height that did not exceed 40 mm. The allograft cutting length was set at 25 mm, and the combined height of the proximal and distal titanium end-discs was 7.5 mm. The outer diameter of the allograft was selected within an approximately 11–13 mm elliptical range to match the end-disc geometry and clinically available structural allograft sizes (Figure 2).

The titanium end-disc incorporated three main features: countersunk screw fixation, open bone-graft filling spaces, and an enlarged bone-contact surface. Each end-disc contained 17° angled screw holes and was fixed to the allograft using M4 × 0.7 screws. The oblique trajectory was selected to increase the length of bone engagement, provide converging resistance against end-disc separation, sliding, and rotation, and avoid the central graft-filling spaces. The screw heads were recessed within the end-disc to prevent protrusion into the adjacent vertebral-endplate contact surface. Multiple open windows allowed graft material to communicate directly with the prepared vertebral endplate rather than being enclosed within a solid titanium component (Figure 2).

### 2.2. Modular Bone-Cutting and End-Disc Assembly System

**Figure 3:**
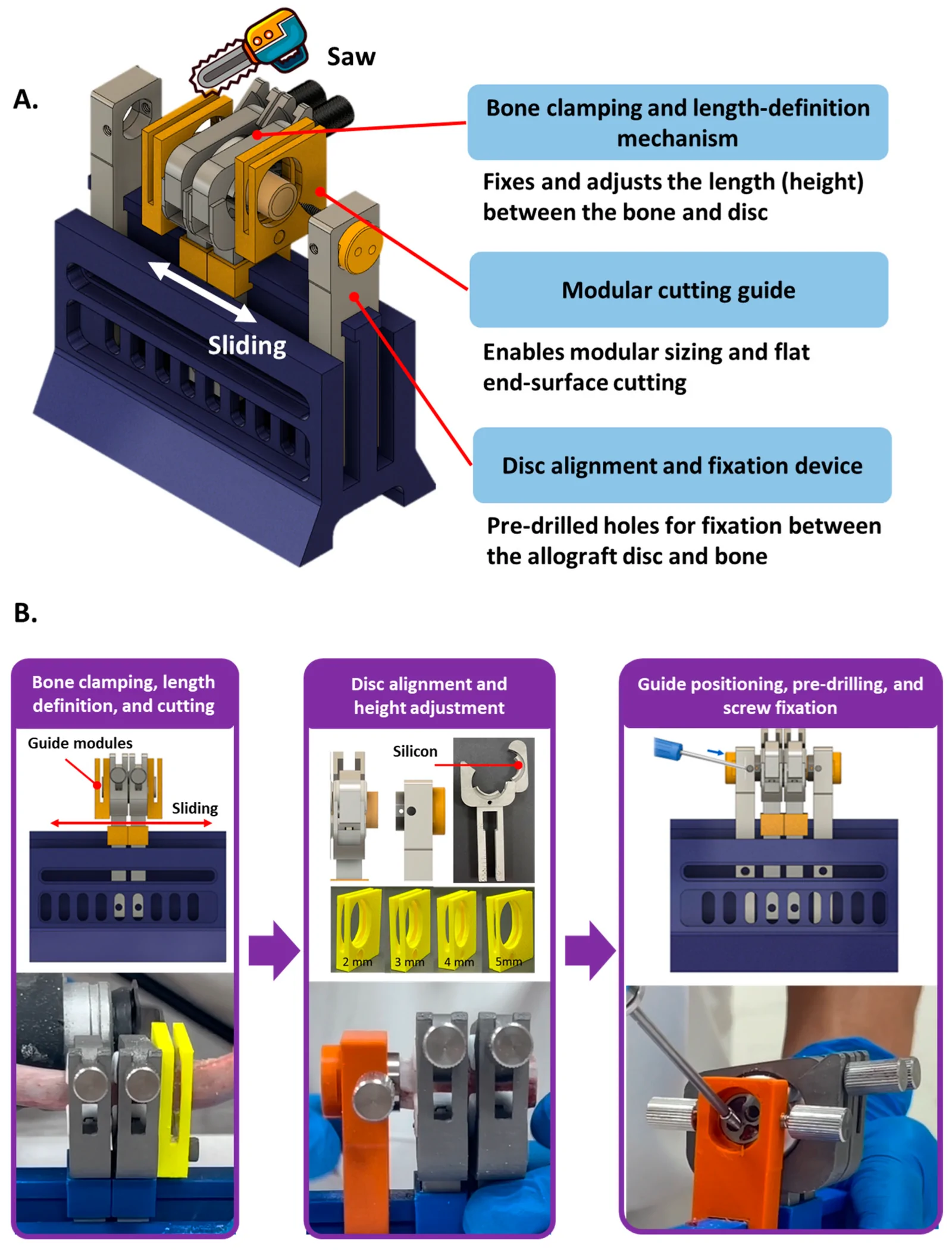
Modular bone-cutting and end-disc assembly auxiliary system. (**A**) The system includes a bone clamping and length-definition mechanism, modular cutting guides, and a disc alignment and fixation device. (**B**) The workflow enables graft clamping, length definition, guided cutting, disc alignment, length (height) adjustment, pre-drilling, and screw fixation for standardized graft–disc construct preparation.

To improve the controllability of allograft cutting length and cut-surface flatness, a modular bone-cutting and end-disc assembly system was designed. The system mainly consisted of a bone clamping and length-defining mechanism, modular cutting guides, and an end-disc alignment and fixation device (Figure 3A).

The bone clamping mechanism was used to secure the proximal and distal ends of the allograft and to control the cutting position and graft length through an adjustable sliding mechanism. A non-slip structure was incorporated into the clamping area to reduce graft slippage during cutting or drilling. The modular cutting guides were designed with different thicknesses (2–5 mm), allowing the operator to select an appropriate guide according to the target graft length and cut along the guide surface. This design was intended to reduce height errors and cut-surface inclination caused by manual trimming.

During preparation, the allograft was first secured in the clamping mechanism on a sterile back table, and the proximal and distal cutting positions were defined using the adjustable sliding mechanism. The graft was trimmed to the target length using the selected guide, aligned with the proximal and distal titanium end-discs, and pre-drilled through the 17° guide holes to create 2.6 mm pilot holes. The fixation screws were then tightened to complete the assembled graft–disc construct. The completed construct was intended to be transferred to the operative field for implantation, whereas the cutting, clamping, alignment, and drilling modules were not intended to be implanted.

### 2.3. Manufacturing and Prototype Validation

The biocompatible titanium alloy Ti6Al4V end-discs and fixation screws were precision-machined by QMS to ensure manufacturing quality and dimensional accuracy. After machining, the main design features, including the screw holes, bone graft filling spaces, bone-contact surfaces, and fixation screws, were inspected to confirm that the components were manufactured according to the intended design.

The bone-cutting and end-disc assembly system were fabricated using different materials according to their functional requirements. The bone clamping mechanism was machined from stainless to provide sufficient clamping rigidity. To prevent slippage of the allograft during clamping and cutting, a silicone sleeve was injection-molded onto the metal clamping surface, as shown by the white region in Figure 3B. This silicone sleeve was designed to increase friction between the graft and the clamp while providing localized flexible contact, thereby reducing the risk of slippage or local damage caused by direct compression of the graft against a hard metal surface.

The modular cutting guides and end-disc fixation device were manufactured by ABS 3D printing (Dimension 1200es SST, Stratasys, Ltd., Minnetonka, MN, USA) to verify the feasibility of the guiding, alignment, and assembly procedures. After fabrication, the complete preparation workflow was tested sequentially, including graft clamping, length definition, guided cutting, end-disc alignment, pre-drilling, and screw locking. This prototype evaluation confirmed that the workflow could be completed. For clinical translation, the prototype instruments would require redesign as reusable sterilizable surgical instruments or validated single-use components, together with cleaning, sterilization, usability, and regulatory evaluation.

### 2.4. Sample Preparation

Porcine ribs were used as an allograft substitute model for preliminary feasibility evaluation. During specimen selection, the long and short axes at the proximal and distal ends of each bone segment were measured. Bone segments with dimensions of approximately 11–13 mm and sufficient length for preparation of a 25 mm graft were selected. Two independent sample groups were prepared: an unassembled native bone group and an assembled graft–disc construct group. The native bone group consisted of bare bone specimens without titanium end-discs, whereas the assembled group was prepared from a different batch of specimens that underwent guided cutting, end-disc alignment, pre-drilling, and screw fixation.

The geometric dimensions of the two independent sample groups are summarized in Table 1. In the native bone group, the dimensional ranges across the four proximal and distal long- and short-axis measurements were 0.8–1.9 mm, with coefficients of variation of 2.50–5.11%. In the assembled graft–disc construct group, the corresponding ranges were 0.5–1.4 mm, with coefficients of variation of 1.42–3.53%. The coefficient of variation was calculated as the standard deviation divided by the mean and expressed as a percentage. These measurements indicated relatively limited geometric variability within each group, although biological variability in bone density, cortical thickness, and internal structure could not be eliminated.

### 2.5. Biomechanical Testing

**Figure 4:**
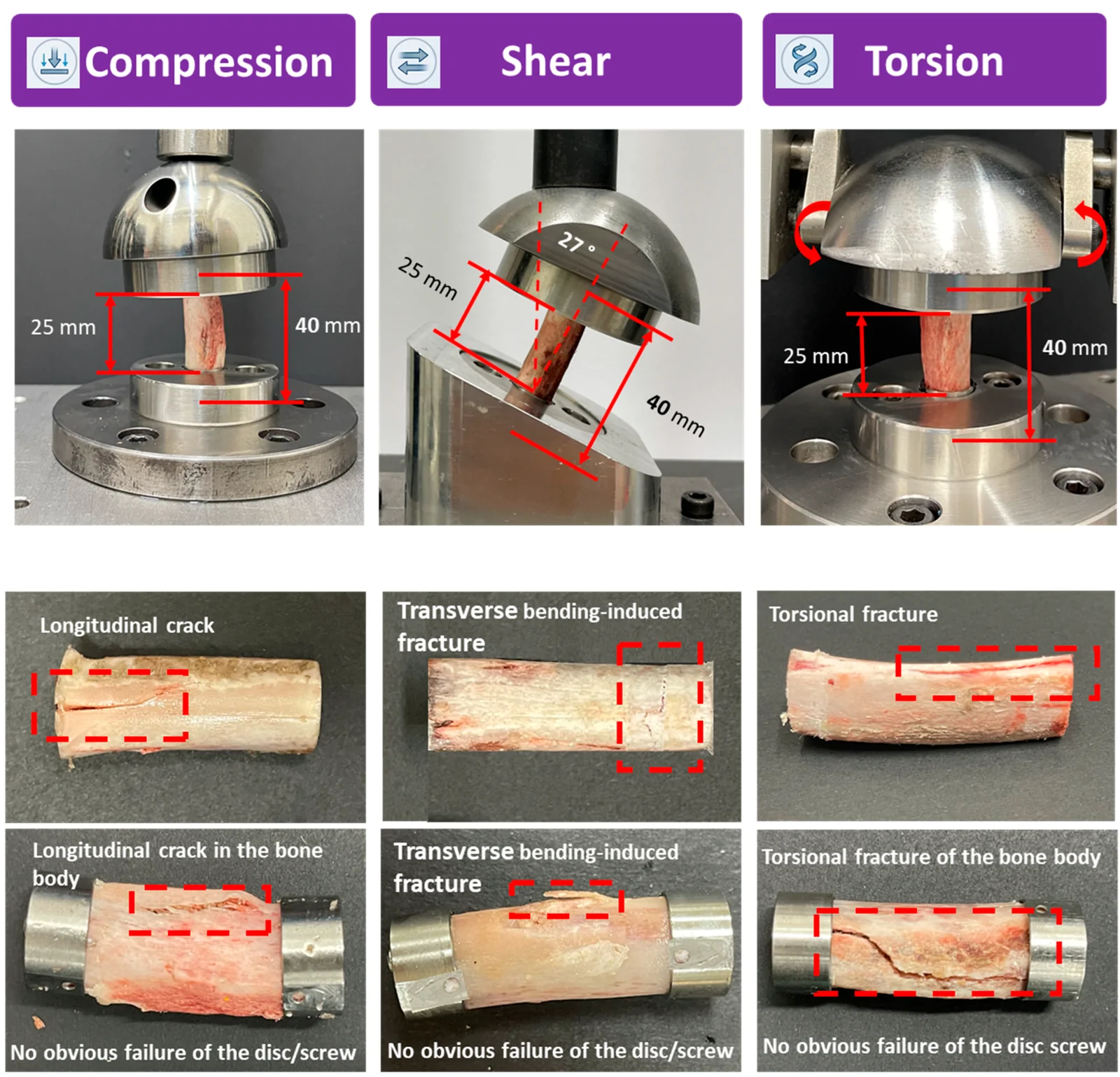
Static biomechanical testing setup and failure modes. Axial compression, compressive shear, and torsion tests were performed to evaluate the initial mechanical stability of the native bone and assembled graft–disc construct groups. After testing, failure in the assembled constructs was mainly observed within the bone body, whereas the titanium end-discs and fixation screws showed no obvious structural failure.

To evaluate the initial structural stability of the titanium end-disc-assisted allograft construct, static axial compression, compressive shear, and torsion tests were performed based on the testing concepts described in ASTM F2077 [19,20]. All tests were conducted using a material testing machine (E3000, Instron ElectroPuls^®^, Instron, Canton, MA, USA). The native bone group and the assembled graft–disc construct group were tested separately, with three specimens included in each group for each loading mode. The purpose of these tests was to compare the mechanical performance of unassembled native bone specimens and assembled graft–disc constructs under different loading conditions, and to determine whether structural damage occurred in the titanium end-discs or fixation screws (Figure 4 upper part).

For the axial compression test, each specimen was placed between upper and lower stainless-steel test fixtures, and a compressive load was applied at a displacement rate of 3 mm/min until specimen failure, a marked decrease in force, or the predefined termination condition was reached. For the compressive shear test, each specimen was positioned in the test fixture at a 27° inclination to generate a shear-loading condition, and compression was applied at the same displacement rate of 3 mm/min. For the torsion test, each specimen was fixed between the upper and lower torsion fixtures. An axial preload of 100 N was first applied, followed by torsional loading at a rate of 60°/min until specimen failure or a marked decrease in torque occurred. During testing, force–displacement and torque–angle curves were recorded (Figure 4 upper part).

For each specimen, stiffness was calculated from the slope of the initial linear region of its force–displacement or torque–angle curve. The yield point was determined individually using the 0.2% offset method rather than by visual identification. A line parallel to the initial linear slope was shifted by an amount corresponding to 0.2% deformation, and its intersection with the experimental curve was defined as the yield point. The corresponding force was recorded as the compressive or shear yield force, whereas the corresponding torque was recorded as the yield torque. The individual yield and stiffness values obtained from the three specimens in each group were then summarized as mean ± standard deviation. No a priori sample-size or statistical power calculation was performed because this was an early-stage feasibility study with three independent specimens per group for each loading mode. Accordingly, the mechanical outcomes were interpreted as preliminary descriptive intergroup comparisons, and no confirmatory inference regarding superiority, equivalence, or non-inferiority was made. After testing, failure modes were documented by imaging and visual inspection, including bone cracking, bone–titanium interface integrity, end-disc integrity, and screw loosening or failure.

## 3. Results

**Figure 5:**
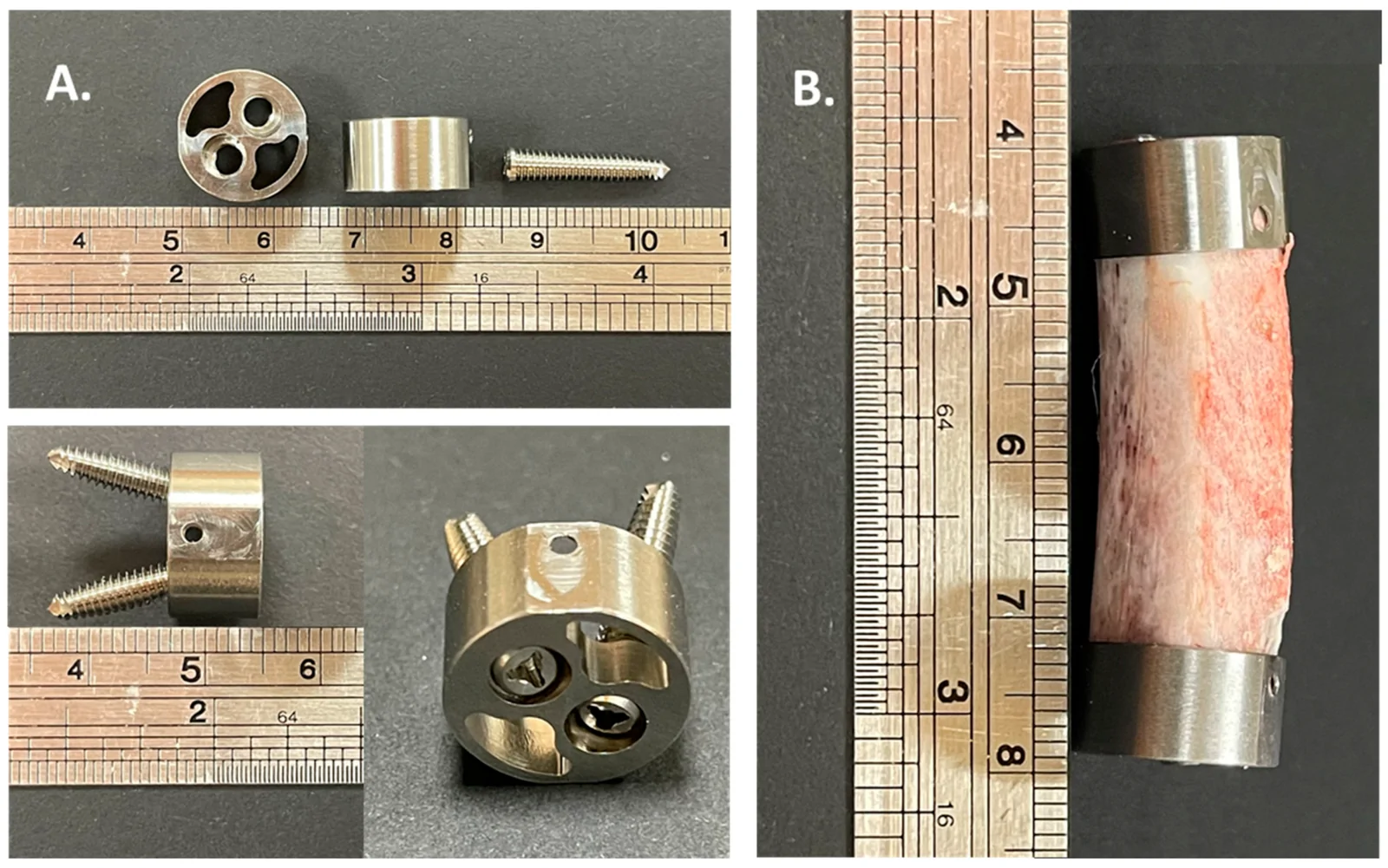
Prototype fabrication and assembled graft–disc construct. (**A**) Fabricated titanium end-disc and fixation screw components, showing screw holes, bone graft filling spaces, and the assembled screw–disc configuration. (**B**) Representative assembled graft–disc construct with proximal and distal titanium end-discs fixed to the bone specimen, achieving the predefined construct height for biomechanical testing.

Prototype fabrication of the titanium end-discs, fixation screws, and modular bone-cutting and end-disc assembly system was successfully completed. The embedded screw holes, bone graft filling spaces, and bone-contact surfaces were clearly identified on the end-disc (Figure 5A). After sequential bone clamping, length definition, cutting guidance, end-disc alignment, pre-drilling, and screw locking, the assembled graft–disc construct was completed with stable fixation of the proximal and distal end-discs to the bone specimen. The final construct height remained within the predefined maximum of 40 mm (Figure 5B). All bone specimens were within the predefined applicable size range of 11–13 mm (Table 1).

**Figure 6:**
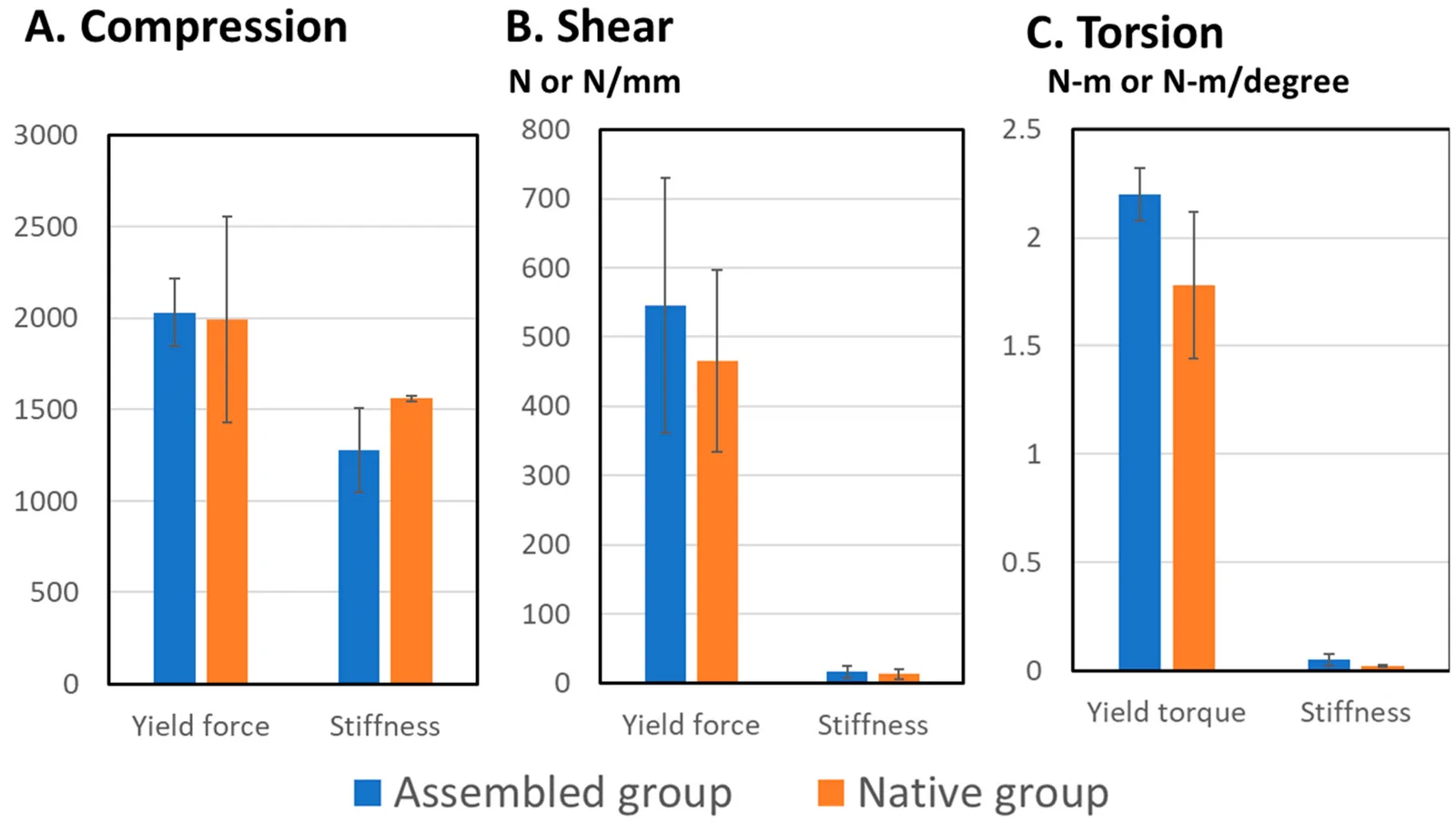
Static biomechanical comparison between native bone and assembled graft–disc constructs. (**A**) Compression, (**B**) shear, and (**C**) torsion testing were performed using ASTM F2077-based configurations. Data are presented as mean ± standard deviation and are interpreted descriptively because only three independent specimens were included in each group for each loading mode. Yield force is reported in N, compression and shear stiffness in N/mm, yield torque in N·m, and torsional stiffness in N·m/degree.

Static axial compression, compressive shear, and torsion tests were performed in the native bone and assembled graft–disc construct groups. Because the groups were prepared from different batches of bone specimens and included only three specimens per loading mode, the results were interpreted as preliminary descriptive intergroup comparisons. Except for axial compressive stiffness, the assembled graft–disc construct showed higher mean values than native bone for compressive yield force, shear yield force, shear stiffness, yield torque, and torsional stiffness (Figure 6).

In axial compression, the compressive yield strength was 2031.0 ± 186.5 N in the assembled graft–disc construct group and 1990.4 ± 562.3 N in the native bone group. The compressive stiffness was lower in the assembled graft–disc construct group than in the native bone group (1280.4 ± 229.7 vs. 1560.0 ± 15.6 N/mm) (Figure 6A). In compressive shear, the assembled graft–disc construct group showed higher shear yield strength (545.6 ± 184.1 vs. 465.2 ± 131.7 N) and shear stiffness (15.9 ± 8.4 vs. 13.3 ± 7.1 N/mm) than the native bone group (Figure 6B). In torsion, the assembled graft–disc construct group also showed higher yield torque (2.20 ± 0.12 vs. 1.78 ± 0.34 N·m) and torsional stiffness (0.049 ± 0.026 vs. 0.021 ± 0.005 N·m/degree) than the native bone group (Figure 6C).

The failure modes after static testing are shown in the lower part of Figure 4. Under axial compression, both groups mainly exhibited longitudinal cracks along the bone axis. Under compressive shear, both groups showed bending-related cracking or localized fracture. Under torsion, torsional cracks or twisting fractures were observed along the bone surface. In the assembled group, failure occurred mainly within the bone body, with no obvious failure at the bone–titanium interface and no evident end-disc damage, screw loosening, or construct separation.

## 4. Discussion

This study presents a modular titanium end-disc fixation and bone-cutting guidance system for standardized allograft preparation in ACCF. Allografts remain clinically attractive because of their osteoconductive potential, availability from bone banks, and possible cost advantages compared with metallic or expandable cages [10,14]. The present system combines titanium end-discs, embedded fixation screws, open graft-filling spaces, and modular cutting guides to reduce operator-dependent variability in graft length, cut-surface flatness, alignment, and fixation. The prototype results support the technical feasibility of preparing a standardized graft–disc construct but do not establish clinical effectiveness.

A principal strength of this study is the integration of a controlled graft-preparation workflow with multidirectional static testing and failure-mode assessment. The modular system is intended for extracorporeal use on a sterile back table rather than implantation. A structural allograft would be selected according to the measured corpectomy defect, secured and trimmed using the modular guides, aligned with the titanium end-discs, pre-drilled, and fixed with countersunk screws. Only the titanium end-discs and fixation screws are intended to remain in the patient as implantable components.

A major design rationale was to improve graft–endplate contact while preserving potential biological fusion pathways. In potential clinical use, residual cartilaginous tissue and localized vertebral-endplate irregularities may be carefully removed to improve contact with the titanium end-discs while preserving the supporting subchondral bone. The construct is intended to occupy the corpectomy defect in the same general position as a structural allograft or cage and is not intended to replace standard supplemental stabilization. Implantation would therefore be followed by anterior cervical plating and screws, or another surgeon-selected fixation method. The open windows in each end-disc are intended to permit graft material to contact the prepared vertebral endplate; however, bone ingrowth, fusion area, vascular access, and osseointegration were not evaluated in the present study.

The assembled graft–disc construct showed higher mean values than native bone for most measured parameters, except axial compressive stiffness. Because the groups were independent and the sample size was limited, these numerical findings are descriptive and should not be interpreted as evidence of superiority, equivalence, or non-inferiority. The observed shear and torsional values may reflect the constraint provided by the oblique screw fixation, but this interpretation requires confirmation in a larger study with controlled bone quality and clinically relevant loading.

The lower compressive stiffness of the assembled graft–disc construct should be interpreted cautiously. This finding may be related to independent bone batches, differences in bone quality, variations in elliptical cross-sectional geometry, small gaps at the end-disc–bone interface, or localized compliance between the titanium end-discs and the bone body. Construct stiffness alone may not determine subsidence risk; load-transfer behavior and vertebral-endplate contact pressure distribution are also important. Further endplate-contact pressure analysis, finite element evaluation, and subsidence testing are therefore required.

Failure-mode assessment showed that damage in the assembled constructs occurred mainly within the bone body. No obvious failure was observed at the bone–titanium interface, titanium end-discs, or fixation screws under the static loading conditions used in this study. These observations indicate that the metallic components were not the obvious initial failure location in this preliminary model; however, fatigue resistance, subsidence behavior, and fixation durability remain to be evaluated.

Future work should include larger prospectively powered studies using matched or randomized human allografts, bone-density characterization, fatigue and subsidence testing, whole cervical spine models with supplemental fixation, endplate-contact pressure analysis, finite element evaluation, porous or lattice surface optimization, and in vitro and in vivo biological assessment.

This study has several limitations. First, porcine ribs were used as an allograft substitute, and their bone quality, cortical thickness, and medullary structure differ from those of clinical human allografts. Second, the native bone and assembled groups were prepared from independent bone batches; geometric screening reduced dimensional variation but could not control bone density, cortical thickness, or internal structure. Third, only three independent specimens were included per group for each loading mode, and no a priori statistical power calculation was performed. The study was therefore not designed for confirmatory statistical inference, and the descriptive findings should not be interpreted as evidence of superiority, equivalence, or non-inferiority. Fourth, only static compression, compressive shear, and torsion were evaluated.

## 5. Conclusions

This study successfully developed a titanium end-disc-assisted allograft reconstruction with a standardized bone-cutting and assembly system for ACCF. Through proximal and distal titanium end-discs, countersunk fixation screws, bone graft filling spaces, and modular cutting guides, assembled graft–disc constructs with controlled graft length, flat cut surfaces, and stable end-disc fixation were successfully prepared. Preliminary static biomechanical testing showed that, except for axial compressive stiffness, the assembled graft–disc construct demonstrated higher mean values than native bone in most mechanical parameters, although the differences were not statistically significant. Failure mainly occurred within the bone body rather than at the titanium end-discs or fixation screws, indicating preliminary mechanical integrity and feasibility of the proposed design. Further fatigue, subsidence, and clinically relevant cervical spine model testing are required to confirm its potential as an auxiliary system for allograft-based ACCF reconstruction.

## Figures and Tables

**Table 1:** Geometric characteristics of native bone specimens and assembled graft–disc constructs.

	Type/No.	Proximal		Distal	
		Long Axis (mm)	Short Axis (mm)	Long Axis (mm)	Short Axis (mm)
**Native bone group**	1	12.7	11.1	12.8	11.2
	2	13.0	11.3	12.8	11.5
	3	13.3	11.9	13.0	11.9
	4	13.0	11.6	12.9	11.9
	5	12.3	11.4	12.8	11.5
	6	12.8	11.7	12.1	11.7
	7	11.4	11.2	12.3	11.1
	8	11.8	11.1	12.3	11.0
	9	12.0	11.2	12.3	11.8
	Mean ± std	12.48 ± 0.6	11.39 ± 0.27	12.59 ± 0.31	11.51 ± 0.32
	Range (mm)	1.9	0.8	0.9	0.9
	CV (%)	5.11	2.5	2.65	2.99
**Assembled graft–disk construct group**	1	13.0	12.4	12.0	11.5
	2	12.7	12.6	12.7	11.5
	3	12.6	12.1	12.0	11.1
	4	12.5	12.0	12.0	12.0
	5	12.5	12.5	12.0	12.0
	6	12.5	12.3	12.5	11.3
	7	12.5	12.0	12.0	11.0
	8	12.8	11.8	13.0	11.8
	9	12.5	11.2	12.8	11.5
	Mean ± std	12.62 ± 0.17	12.10 ± 0.4	12.33 ± 0.39	11.52 ± 0.34
	Range (mm)	0.5	1.4	1.0	1.0
	CV (%)	1.42	3.53	3.37	3.12

## Data Availability

The raw data supporting the conclusions of this article will be made available by the authors on request.
